# JOIN trial: treatment outcome and recovery status of peripheral sensory neuropathy during a 3-year follow-up in patients receiving modified FOLFOX6 as adjuvant treatment for stage II/III colon cancer

**DOI:** 10.1007/s00280-019-03957-5

**Published:** 2019-09-23

**Authors:** Takayuki Yoshino, Masahito Kotaka, Katsunori Shinozaki, Tetsuo Touyama, Dai Manaka, Takanori Matsui, Kiyoshi Ishigure, Junichi Hasegawa, Keiji Inoue, Yoshinori Munemoto, Akinori Takagane, Hiroshi Ishikawa, Hideyuki Ishida, Yutaka Ogata, Koji Oba, Koichi Goto, Junichi Sakamoto, Yoshihiko Maehara, Atsushi Ohtsu

**Affiliations:** 1 Department of Gastroenterology and Gastrointestinal Oncology, National Cancer Center Hospital East, 6-5-1 Kashiwanoha, Kashiwa, 277-8577 Japan; 2 Gastrointestinal Cancer Center, Sano Hospital, Kobe, 655-0031 Japan; 3 Division of Clinical Oncology, Hiroshima Prefectural Hospital, Hiroshima, 734-8530 Japan; 4 Department of Surgery, Nakagami Hospital, Okinawa, 904-2195 Japan; 5 Department of Surgery, Gastrointestinal Center, Kyoto Katsura Hospital, Kyoto, 615-8256 Japan; 6 Department of Gastroenterological Surgery, Aichi Cancer Center Aichi Hospital, Aichi, 444-0011 Japan; 7 Department of Surgery, Konan Kosei Hospital, Konan, Aichi 483-8704 Japan; 8 Department of Surgery, Osaka Rosai Hospital, Osaka, 591-8025 Japan; 9 Surgery Nagasaki Harbor Medical Center City Hospital, Nagasaki, 850-8555 Japan; 10 Department of Surgery, Fukui-ken Saiseikai Hospital, Fukui, 918-8503 Japan; 11 Department of Surgery, Hakodate Goryoukaku Hospital, Hokkaido, 040-8611 Japan; 12 Department of Gastrointestinal Surgery, Sasebo City General Hospital, Nagasaki, 857-8511 Japan; 13 Department of Digestive Tract and General Surgery, Saitama Medical Center, Saitama Medical University, Kawagoe, 350-8550 Japan; 14 Cancer Center, Kurume University Hospital, Fukuoka, 830-0011 Japan; 15 Department of Biostatistics, School of Public Health, Graduate School of Medicine, and Interfaculty Initiative in Information Studies, The University of Tokyo, Tokyo, 113-8655 Japan; 16 Department of Thoracic Oncology, National Cancer Center Hospital East, Kashiwa, 277-8577 Japan; 17 Tokai Central Hospital, Kakamigahara, 504-8601 Japan; 18 Japanese Foundation for Multidisciplinary Treatment of Cancer, Tokyo, 136-0071 Japan

**Keywords:** Modified FOLFOX6, Long-term peripheral sensory neuropathy, Oxaliplatin, Colon cancer, Efficacy

## Abstract

**Purpose:**

Adjuvant FOLFOX therapy is an established standard-of-care for resected colon cancer. Peripheral sensory neuropathy (PSN) is regarded as the major toxicity issue related to FOLFOX therapy. There have been a few reports on the recovery status from PSN thereafter. JOIN trial investigated the tolerability and efficacy of adjuvant modified FOLFOX6 (mFOLFOX6) in Japanese patients with stage II/III colon cancer.

**Methods:**

Twelve cycles of mFOLFOX6 were given to patients with stage II/III curatively resected colon cancer. Treatment outcomes, including disease-free survival (DFS), relapse-free survival (RFS), overall survival (OS), and recovery status of PSN during 3-year follow-up, were investigated.

**Results:**

Of the 882 patients enrolled from 2010 to 2012, 864 were eligible for the efficacy analyses. Three-year DFS, RFS, and OS were favorable in 92.1, 92.8, and 97.4% of stage II patients; 76.4, 77.9, and 93.8% of stage IIIA/B; and 61.6, 62.7, and 85.9% of stage IIIC, respectively. The cumulative incidence of PSN during treatment was 47.8% in grade 1 (G1), 30.3% in G2, and 5.8% in G3. For those with G3 PSN during treatment, there was gradual recovery in 1.1% of patients at 12 months after enrollment, 0.5% at 24 months, and 0.2% at 36 months. However, G1 or G2 residual PSN after 3 years was observed in 21.0% (18.7%, G1; 2.3%, G2).

**Conclusions:**

Adjuvant mFOLFOX6 therapy was effective and well tolerated in patients with stage II/III colon cancer. Most patients recovered from G3 PSN related to oxaliplatin, but approximately 20% of patients had G1 or G2 PSN at 3-year follow-up.

**Electronic supplementary material:**

The online version of this article (10.1007/s00280-019-03957-5) contains supplementary material, which is available to authorized users.

## Introduction

Six months of adjuvant oxaliplatin-based 5-fluorouracil (5-FU), leucovorin and oxaliplatin (FOLFOX) chemotherapy following surgery is the standard care for patients with stage III colon cancer as well as for patients with high-risk stage II colon cancer who have risk factors for recurrence that are associated with a relatively poor prognosis, such as T4 status, poorly differentiated histology, vascular invasion, ileus, < 12 lymph nodes examined, and neural invasion, as recommended by several treatment guidelines [1–3].

FOLFOX4 regimen has proven efficacy in the adjuvant treatment of resected stage II and stage III colon cancers, as demonstrated by the pivotal MOSAIC [4, 5] and MASCOT [6] trials conducted in Western and Asian patient populations, respectively. More recently, FOLFOX4 has frequently been substituted by the modified FOLFOX6 (mFOLFOX6) regimen in the adjuvant setting [7, 8], which is easier to administer, and mFOLFOX6 has been shown to be tolerable in the adjuvant treatment of Japanese patients with resected stage II and stage III colon cancer in the JOIN (JFMC41-1001-C2) trial [9].

Many patients, however, develop peripheral sensory neuropathy (PSN) by the standard 6 months, 12 cycles of mFOLFOX6 administration, leading to treatment discontinuations [10–14]. PSN can be troublesome in daily life and problems usually persist long after treatment has finished. In the key trials of adjuvant oxaliplatin-based therapy, the incidences of grade ≥ 3 PSN due to FOLFOX4 therapy during treatment were 12.4% and 5.7% for the MOSAIC and Asian MASCOT trials, respectively [4–6]. In the adjuvant US NSABP C-08 trial [7] and Japanese JOIN trial [9] in which patients received the mFOLFOX6 regimen, the incidences of grade ≥ 3 PSN at the end of the studies were 14.4% and 5.8%, respectively. During a 3-year follow-up in patients receiving FOLFOX4 in the MOSAIC trial [5], the recovery status of PSN for any grade and grade 3 PSNs was reported as having a frequency of 18.1 and 0.6%, respectively. No trial has revealed the PSN recovery rate from mFOLFOX6, particularly in Asian patients. In addition, 6 months of FOLFOX treatment even in the post-IDEA collaboration era is a standard adjuvant treatment in patients with curatively resected stage II/III colon cancer [3]. The results of the JOIN trial for safety during the treatment course and the treatment compliance have been reported elsewhere [9]. Here, we report the treatment outcomes including disease-free survival (DFS), relapse-free survival (RFS), overall survival (OS), and recovery rate from PSN during a 3-year follow-up.

## Patients and methods

### Study design

The study design of the JOIN study has been reported previously [9]. Briefly, the JOIN trial is a single-arm, multi-center, large-scale clinical trial across Japan to confirm the tolerability of adjuvant mFOLFOX6 in patients with curatively resected stage II/III colon cancer (UMIN ID: UMIN000004443).

### Treatments

The study treatment was mFOLFOX6 therapy (L-OHP, 85 mg/m^2^; 1-LV, 200 mg/m^2^; 5-FU bolus, 400 mg/m^2^; and 5-FU infusion, 2400 mg/m^2^), with a total of 12 courses being administered at 2-week intervals. Further chemotherapy was not given until recurrence after completion of the scheduled therapy.

### Endpoints

The primary endpoints were the incidence of PSN persisting for ≥ 8 days that interfered with activities of daily living and with an incidence of ≥ grade 3 allergic reactions/anaphylaxis (AR). Secondary endpoints were DFS, RFS, OS, time to treatment failure, adverse events (AEs), comparison of PSN between patients with or without receiving prophylactic therapy, recovery status of PSN during the 3-year follow-up period, the treatment completion rate, the relative dose intensity (RDI), and the number of lymph-node metastases and number of dissected lymph nodes in relation to the prognosis. DFS was defined as the time from enrollment to relapse, secondary primary colorectal cancer, or death, whichever occurred first. RFS was defined as the time from enrollment to relapse, or death, whichever occurred first. OS was measured from the time of enrollment until death from any cause.

AEs were evaluated according to the Common Terminology Criteria for Adverse Events (CTCAE) Version 4.0. However, PSN was evaluated by following the NCI-CTC Version 1.0, 2.0 and CTCAE Version 3.0.

### Statistical analysis

Descriptive statistics were calculated as the number of patients and percentage for categorical baseline characteristics and mean with a range for continuous baseline characteristics. Three-year DFS, RFS, and OS rates were estimated using the Kaplan–Meier method. Greenwood’s formula was applied for calculation of the 95% confidence interval (95% CI) of the 3-year DFS, RFS, and OS. The multivariate Cox proportional hazard model was applied to evaluate the prognostic value of patient baseline characteristics for DFS, RFS, and OS. Covariates were selected using the stepwise method with an inclusion criterion of *p *< 0.20. The proportion of recovery status of PSN during the 3-year follow-up period was calculated by the number of PSN in the total efficacy population at each follow-up. All statistical analyses were performed using SAS Version 9.3 (SAS Institute, Cary, NC, USA).

## Results

### Patient population and baseline characteristics

**Fig. 1:**
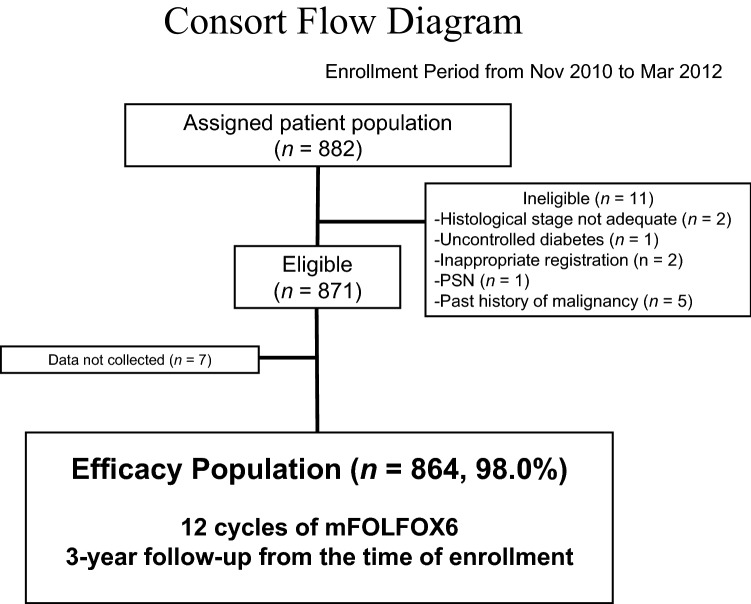
CONSORT diagram. Between November 2010 and March 2012, 882 patients were enrolled at 198 institutions. Among these 882 patients, 11 were ineligible, as previously reported. Of the remaining 871 eligible patients, 864 patients (98.0%) for whom the treatment status was fixed were included in the efficacy analysis

Between November 2010 and March 2012, 882 patients were enrolled at 198 institutions. Among these 882 patients, 11 were ineligible, as previously reported [9]. Of the remaining 871 eligible patients, 864 patients (98.0%) for whom the treatment status was fixed with a median follow-up of 3 years as of October 30, 2015 via an electronic data capture system (Viedoc^®^, PCG Solutions, Uppsala, Sweden) with central monitoring were included in the efficacy analysis (Fig. 1). The characteristics of these patients are shown in Table 1. Baseline patient characteristics were as follows: median age, 64 years; male, 53.8%; PS 0, 93.8%; stage II/IIIA/IIIB/IIIC by TNM Classification, 7th edition, 18.5/7.3/52.5/21.6%; and lymph nodes examined, < 12/≥ 12/unknown: 17.2/82.5/0.2%, respectively.

**Table 1:** Patient characteristics

*n* (%)	864
Male/female	465/399 (53.8/46.2)
Median age (range)	64 (21–83)
PS:	810/54
0/1	(93.8/6.2)
Stage (TNM 7th)^a^:	100/34/26/63/454/187
IIA/IIB/IIC/IIIA/IIIB/IIIC	(11.6/3.9/3.0/7.3/52.5/21.6)
Number of lymph nodes examined	2/149/713
Unknown/1–11/≥ 12	(0.2/17.2/82.5)
Number of positive lymph nodes	160/424/280
0/1–3/≥ 4	(18.5/49.1/32.4)

^a^TNM classification of malignant tumors, 7th edition

### Treatment outcome

**Fig. 2:**
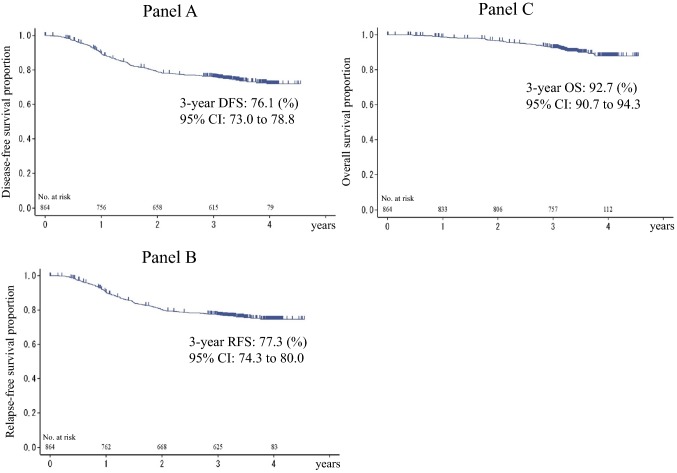
Kaplan–Meier curves for **a** DFS, **b** RFS, and **c** OS in the overall population. *DFS* disease-free survival, *RFS* relapse-free survival, *OS* overall survival, *95% CI* 95% confidence interval

**Fig. 3:**
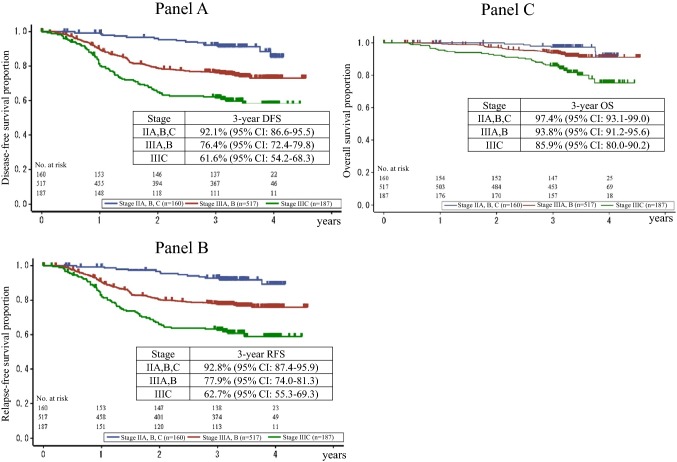
Kaplan–Meier curves for **a** DFS, **b** RFS and **c** OS in the overall population stratified by stage in the TNM Classification of Malignant Tumors, 7th edition. *DFS* disease-free survival, *RFS* relapse-free survival, *OS* overall survival, *95% CI* 95% confidence interval

Three-year DFS, RFS, and OS in the overall efficacy population were 76.1% (95% CI 73.0–78.8), 77.3% (95% CI 74.3–80.0), and 92.7% (95% CI 90.7–94.3), respectively (Fig. 2). Favorable 3-year DFS, RFS, and OS were 92.1, 92.8, and 97.4% in stage II patients, while these were 76.4, 77.9, and 93.8% in stage IIIA/B; and 61.6, 62.7, and 85.9% in stage IIIC, respectively (Fig. 3). The main recurrent sites were liver (7.6%), lung (7.3%), and lymph nodes (5.2%). In multivariate Cox regression analysis, tumor histology, venous invasion, and lymph-node metastatic ratio were statistically significant prognostic factors for DFS, RFS, and OS (Table 2), while the tumor location was not significant, although OS in left-sided primary tumors was better than that in right-sided ones with statistical non-significance (Supplementary Fig. 1).

**Table 2:** Multivariate analysis of risk factors for DFS, RFS and OS

	DFS			RFS			OS		
Risk factor	HR^a^	95% CI^b^	*P*	HR	95% CI	*P*	HR	95% CI	*P*
*T*									
SM, MP, SS, A	1.000			1.000			1.000		
SE, SI, AI	1.386	1.041–1.845	0.0255	1.441	1.073–1.937	0.0152	1.406	0.878–2.251	0.1556
Histology									
Pap, tub	1.000			1.000			1.000		
Por, muc, sig	1.570	1.028–2.398	0.0367	1.569	1.017–2.419	0.0416	2.860	1.619–5.051	0.0003
*N* ^*c*^									
*N*0	1.000			1.000			1.000		
*N*1	1.793	0.784–4.100	0.1663	1.375	0.522–3.618	0.5194	1.395	0.348–5.592	0.6380
*N*2, *N*3	1.624	0.658–4.008	0.2924	1.309	0.464–3.693	0.6104	1.412	0.306–6.525	0.6587
v									
v0	1.000			1.000			1.000		
v1, v2, v3	1.347	0.954–1.903	0.0907	1.472	1.017–2.129	0.0402	1.957	1.032–3.777	0.0398
LN ratio (%)									
1–4	1.000			1.000			1.000		
5–11	1.469	0.719–3.001	0.2918	2.011	0.855–4.732	0.1094	1.326	0.382–4.607	0.6572
12–22	1.920	0.938–3.930	0.0742	2.741	1.165–6.444	0.0208	1.563	0.446–5.483	0.4854
22 <	2.924	1.380–6.198	0.0051	3.930	1.619–9.539	0.0025	2.838	0.762–10.56	0.1199
Tumor location									
Right-sided							1.000		
Left-sided							0.633	0.398–1.006	0.0530

^a^Hazard ratio

^b^Confidence interval

^c^Japanese classification of colorectal carcinoma 7th ed

### Recovery status of PSN

**Fig. 4:**
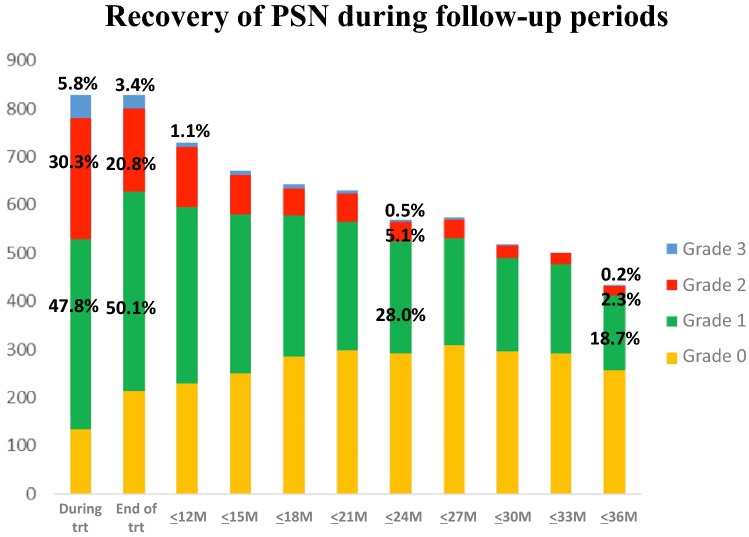
Recovery status of PSN during follow-up periods. *PSN* peripheral sensory neuropathy, *trt* treatment

The cumulative incidence of PSN during treatment was 47.8% grade 1, 30.3% grade 2, and 5.8% grade 3, respectively (Fig. 4). Grade 3 PSN appeared to gradually recover from 5.8% to 1.1%, 0.5%, and 0.2% at 12 months, 24 months, and 36 months after enrollment, respectively. However, grade 1 or grade 2 PSNs after 3-year follow-up were observed in 21.0% of patients (18.7% in grade 1 and 2.3% in grade 2). The transition from each grade of PSN during study treatment (from grade 1, grade 2, and grade 3) is shown in Supplementary Fig. 2.

## Discussion

The JOIN trial results confirm the efficacy and safety of adjuvant mFOLFOX6 in over 800 patients with stage II/III curatively resected colon cancer among approximately 200 community hospitals in Japan. Approximately 6% of Asian patients only showed grade 3 or higher PSN in the JOIN and MASCOT, while 12–14% patients did in the Western trials such as MOSAIC and NSABP C-08 [5–9].

The PSN during treatment was the highest and gradually recovered during the 3-year follow-up period. In addition, it was confirmed that approximately 20% of patients had grade 1 or grade 2 PSN from the 3-year PSN follow-up, which is consistent with that of FOLFOX4 [5]. PSN caused by oxaliplatin, either FOLFOX4 or mFOLFOX6, is a common issue worldwide. However, the remaining PSN may be influenced by factors other than oxaliplatin. In the NSABP C-07 trial [15] comparing weekly bolus fluorouracil and leucovorin (FL) therapy with or without oxaliplatin, the PSN for some patients (less than 5%) remained in the FL group at 12 months of follow-up, suggesting that the PSN may occur due to factors other than oxaliplatin.

Three-year DFS and OS in the JOIN trial were 76.1 and 92.7%, respectively. Stratified by stage, 3-year DFS and OS were 92.1 and 97.4% in stage II, 76.4 and 93.8% in stage IIIA/B, 61.6, and 85.9% in stage IIIC, respectively. Three-year DFS in other recent clinical trials conducted in Japan for stage II/III or stage III colon cancer were reported to range from 70.0 to 86.2%, while those in 3-year OS were reported to range from 92.7 to 98.0%, which is consistent with those in the JOIN trial (Supplementary Table) [16–22].

Based on the results of Japanese clinical trials including the JOIN trial, the survival rate of patients with stage II/III colon cancer appeared to be relatively higher in Japan than in Western countries (Supplementary Table) [16–22]. This difference might be largely due to the lower percentage of patients with < 12 nodes examined as well as the Japanese D3 lymph-node dissection procedure, given that the number of lymph nodes examined after surgical resection was shown to correlate with survival [23]. In fact, the percentage of patients with < 12 nodes examined in Japanese trials in patients with stage II and III colon cancer such as JOIN, SACURA, and ACTS-CC trials was substantially lower than that in the NSABP C-07 trial, which enrolled subjects during the same period (17–26% vs. 41%, respectively). However, recently, a comparison of Japanese D3 lymph-node dissection and European complete mesocolic excision (CME) with central vascular ligation has been reported [24], and favorable outcomes of CME compared with conventional Western-style colon resection have also been reported from Western countries [25]. These findings imply that the gap in the survival of patients with stage II/III colon cancer between Japan and Western countries is closing, suggesting that Japanese outcomes might be applicable to the West. Considering the recent improved survival rate of patients with stage II/III colon cancer worldwide, we should reconsider the relatively high oxaliplatin-associated toxicity which could be weighed against the expectation of lower absolute treatment benefit in patients with a low risk of recurrence.

Recent IDEA collaboration reported 3 months adjuvant chemotherapy significantly reduced the rate of any grade PSN, compared with 6 months, without compromising efficacy in patients with low-risk stage III colon cancer [26, 27]. In addition, the ACHIEVE trial, the part of IDEA collaboration, reported the incidence of any grade PSN lasting for 3 years was significantly lower for CAPOX (capecitabine plus oxaliplatin) than mFOLFOX6, suggesting that 3 months of CAPOX therapy may be the most appropriate option in low-risk patients [28], although ACHIEVE results need to be interpreted within the IDEA combined analysis as well as in terms of the reproducibility of the results across all trials. Therefore, 6 months of FOLFOX treatment even in the post-IDEA collaboration era is a standard adjuvant treatment in patients with curatively resected stage II/III colon cancer [3]. As the efficacy and safety of 6-month adjuvant mFOLFOX6 in Japanese patients with stage II/III colon cancer was confirmed in the JOIN trial, we moved forward to conduct two-phase three trials called the ACHIEVE trial for stage III and the ACHIEVE-2 trial for high-risk stage II [29, 30], which led to the first Japanese participation in the IDEA Collaboration [26, 27].

## Conclusion

Six-month adjuvant mFOLFOX6 in patients with stage II/III colon cancer is effective and safe. Most patients successfully recovered from grade 3 PSN related to oxaliplatin, but approximately 20% of patients had grade 1 or grade 2 PSN at the 3-year follow-up.

## Electronic supplementary material

Below is the link to the electronic supplementary material.

Supplementary material 1 (PPTX 177 kb). Kaplan–Meier curves for **a** DFS, **b** RFS and **c** OS in the overall population stratified by primary tumor location. *DFS* disease-free survival, *RFS* relapse-free survival, *OS* overall survival, *95% CI* 95% confidence interval

Supplementary material 2 (PPTX 211 kb). Recovery status of each grade PSN (from **a** grade 1, **b** grade 2 and **c** grade 3) during study treatment during follow-up periods. *PSN* peripheral sensory neuropathy, *trt* treatment

Supplementary material 3 (DOCX 12 kb)

## References

[1] Labianca R, Nordlinger B, Beretta GD (2013). Early colon cancer: ESMO clinical practice guidelines for diagnosis, treatment and follow-up. Ann Oncol.

[2] Watanabe T, Muro K, Ajioka Y (2017). Japanese society for cancer of the colon and rectum (JSCCR) guidelines 2016 for the treatment of colorectal cancer. Int J Clin Oncol.

[3] NCCN guidelines for patients: colon cancer version 4 (2018) www.nccn.org. Accessed 5 Nov 2018

[4] Andre T, Boni C, Mounedji-Boudiaf L (2004). Oxaliplatin, fluorouracil, and leucovorin as adjuvant treatment for colon cancer. N Engl J Med.

[5] Andre T, Boni C, Navarro M (2009). Improved overall survival with oxaliplatin, fluorouracil, and leucovorin as adjuvant treatment in stage II or III colon cancer in the MOSAIC trial. J Clin Oncol.

[6] Lee P-H, Park Y-S, Ji J-F, Fu Y-T, Ratanatharathorn V (2009). Safety and tolerability of FOLFOX4 in the adjuvant treatment of colon cancer in Asian patients: the MASCOT study. Asia-Pac J Clin Oncol.

[7] Allegra CJ, Yothers G, O’Connell MJ (2009). Initial safety report of NSABP C-08: a randomized phase III study of modified FOLFOX6 with or without bevacizumab for the adjuvant treatment of patients with stage II or III colon cancer. J Clin Oncol.

[8] Allegra CJ, Yothers G, O’Connell MJ (2011). Phase III trial assessing bevacizumab in stages II and III carcinoma of the colon: results of NSABP protocol C-08. J Clin Oncol.

[9] Kotaka M, Yoshino T, Oba K (2015). Initial safety report on the tolerability of modified FOLFOX6 as adjuvant therapy in patients with curatively resected stage II or III colon cancer (JFMC41-1001-C2: JOIN trial). Cancer Chemother Pharmacol.

[10] Kidwell KM, Yothers G, Ganz PA (2012). Long-term neurotoxicity effects of oxaliplatin added to fluorouracil and leucovorin as adjuvant therapy for colon cancer: results from National Surgical Adjuvant Breast and Bowel Project trials C-07 and LTS-01. Cancer.

[11] Mols F, Beijers T, Lemmens V, van den Hurk CJ, Vreugdenhil G, van de Poll-Franse LV (2013). Chemotherapy-induced neuropathy and its association with quality of life among 2- to 11-year colorectal cancer survivors: results from the population-based PROFILES registry. J Clin Oncol.

[12] Pachman DR, Qin R, Seisler DK (2015). Clinical course of oxaliplatin-induced neuropathy: results from the randomized phase III trial N08CB (alliance). J Clin Oncol.

[13] Pietrangeli A, Leandri M, Terzoli E, Jandolo B, Garufi C (2006). Persistence of high-dose oxaliplatin-induced neuropathy at long-term follow-up. Eur Neurol.

[14] Tofthagen C, Donovan KA, Morgan MA, Shibata D, Yeh Y (2013). Oxaliplatin-induced peripheral neuropathy’s effects on health-related quality of life of colorectal cancer survivors. Support Care Cancer.

[15] Land SR, Kopec JA, Cecchini RS, Ganz PA, Wieand HS, Colangelo LH, Murphy K, Kuebler JP, Seay TE, Needles BM, Bearden JD, Colman LK, Lanier KS, Pajon ER, Cella D, Smith RE, O’Connell MJ, Costantino JP, Wolmark N (2007). Neurotoxicity from oxaliplatin combined with weekly bolus fluorouracil and leucovorin as surgical adjuvant chemotherapy for stage II and III colon cancer: NSABP C-07. J Clin Oncol.

[16] Kotani D, Kuboki Y, Yoshino T (2016). Adjuvant chemotherapy for colon cancer: guidelines and clinical trials in Japan. Curr Colorectal Cancer Rep.

[17] Kondo K, Sadahiro S, Tsuchiya T, Sasaki K, Katsumata K, Nishimura G, Kakeji Y, Baba H, Kodaira S, Saji S (2012) Phase III trial of treatment duration for oral uracil and tegafur/leucovorin adjuvant chemotherapy for patients with stage IIB/III colon cancer: results of JFMC33-0502. ESMO 2012 Abstract #552P 10.1093/annonc/mdv358 PMC4621030 26347106

[18] Yamaguchi S, Kunieda K, Sato T, Naomoto Y, Kobayashi M, Ogata Y, Furuhata T, Takii Y, Kusunoki M, Maehara Y, Koda K, Okuno K, Ohno M, Mishima H, Sadahiro S, Hamada C, Sakamoto J, Saji S, Tomita N (2016) Phase III trial of 24 weeks vs. 48 weeks capecitabine adjuvant chemotherapy for patients with stage III colon cancer: final results of JFMC37-0801. ESMO 2016 Abstract #469PD

[19] Yoshida M, Ishiguro M, Ikejiri K, Mochizuki I, Nakamoto Y, Kinugasa Y, Takagane A, Endo T, Shinozaki H, Takii Y, Mochizuki H, Kotake K, Kameoka S, Takahashi K, Watanabe T, Watanabe M, Boku N, Tomita N, Nakatani E, Sugihara K (2014). ACTS-CC study group. S-1 as adjuvant chemotherapy for stage III colon cancer: a randomized phase III study (ACTS-CC trial). Ann Oncol.

[20] Shimada Y, Hamaguchi T, Mizusawa J, Saito N, Kanemitsu Y, Takiguchi N, Ohue M, Kato T, Takii Y, Sato T, Tomita N, Yamaguchi S, Akaike M, Mishima H, Kubo Y, Nakamura K, Fukuda H, Moriya Y (2014). Randomised phase III trial of adjuvant chemotherapy with oral uracil and tegafur plus leucovorin versus intravenous fluorouracil and levofolinate in patients with stage III colorectal cancer who have undergone Japanese D2/D3 lymph node dissection: final results of JCOG0205. Eur J Cancer.

[21] Hamaguchi T, Shimada Y, Mizusawa J, Kinugasa Y, Kanemitsu Y, Ohue M, Fujii S, Takiguchi N, Yatsuoka T, Takii Y, Ojima H, Masuko H, Kubo Y, Mishima H, Yamaguchi T, Bando H, Sato T, Kato T, Nakamura K, Fukuda H, Moriya Y (2018). Capecitabine versus S-1 as adjuvant chemotherapy for patients with stage III colorectal cancer (JCOG0910): an open-label, non-inferiority, randomised, phase 3, multicentre trial. Lancet Gastroenterol Hepatol.

[22] Kajiwara Y, Ishiguro M, Teramukai S, Matsuda C, Fujii S, Kinugasa Y, Nakamoto Y, Kotake M, Sakamoto Y, Kurachi K, Maeda A, Komori K, Tomita N, Shimada Y, Takahashi K, Kotake K, Watanabe M, Mochizuki H, Sugihara K, SACURA Study Group (2018). A randomized phase III trial of 1-year adjuvant chemotherapy with oral tegafur-uracil (UFT) vs surgery alone in stage II colon cancer: SACURA trial. Eur J Cancer.

[23] Chang GJ, Rodriguez-Bigas MA (2007). Lymph node evaluation and survival after curative resection of colon cancer: systematic review. J Natl Cancer Inst.

[24] Nicholas PW, Kobayashi H, Takahashi K (2012). Understanding optimal colonic cancer surgery: comparison of Japanese D3 resection and European complete mesocolic excision with central vascular ligation. J Clin Oncol.

[25] Bertelsen CA, Neuenschwander AU, Jansen JE (2015). Disease-free survival after complete mesocolic excision compared with conventional colon cancer surgery: a retrospective, population-based study. Lancet Oncol.

[26] Andre T, Iveson T, Labianca R (2013). The IDEA (International Duration Evaluation of Adjuvant Chemotherapy) collaboration: prospective combined analysis of phase III trials investigating duration of adjuvant therapy with the FOLFOX (FOLFOX4 or Modified FOLFOX6) or XELOX (3 versus 6 months) regimen for patients with stage III Colon Cancer: trial design and current status. Curr Colorectal Cancer Rep.

[27] Grothey A, Sobrero AF, Shields AF (2018). Duration of adjuvant chemotherapy for stage III colon cancer. N Engl J Med.

[28] Takeuchi S, Yoshino T, Yamanaka T (2018). Long-term effect of peripheral sensory neuropathy (PSN) of 3 or 6 months oxaliplatin-based adjuvant chemotherapy for stage III colon cancer: ACHIEVE as part of the IDEA collaboration. Age.

[29] Yoshino T, Watanabe T, Mori M, et al (2014) Two phase III studies comparing 6 months of either mFOLFOX6 or XELOX with 3 months of the same regimen as adjuvant chemotherapy in patients with completely resected stage III colon cancer (ACHIEVE) or high-risk stage II colon cancer (ACHIEVE-2). ASCO 2014 Abstract #TPS 3655

[30] Yoshino T, Yamanaka T, Kotaka M, et al (2017) Efficacy of 3 versus 6 months of oxaliplatin-based adjuvant chemotherapy for stage III colon cancer (CC): Results from phase III ACHIEVE trial as part of the International Duration Evaluation of Adjuvant therapy (IDEA) collaboration. ESMO 2017 Abstract #LBA 24

