# Supplementary material for: JOIN trial: treatment outcome and recovery status of peripheral sensory neuropathy during a 3-year follow-up in patients receiving modified FOLFOX6 as adjuvant treatment for stage II/III colon cancer
Source: Cancer Chemother Pharmacol. 2019 Sep 23;84(6):1269–77. doi: 10.1007/s00280-019-03957-5 (PMC6820589; doi:10.1007/s00280-019-03957-5)
Supplement: Supplementary file 1 — Supplementary material 1 (PPTX 177 kb). Kaplan–Meier curves for a DFS, b RFS and c OS in the overall population stratified by primary tumor location. DFS disease-free survival, RFS relapse-free survival, OS overall survival, 95% CI 95% confidence interval [file 280_2019_3957_MOESM1_ESM.pptx]

## Slide 1
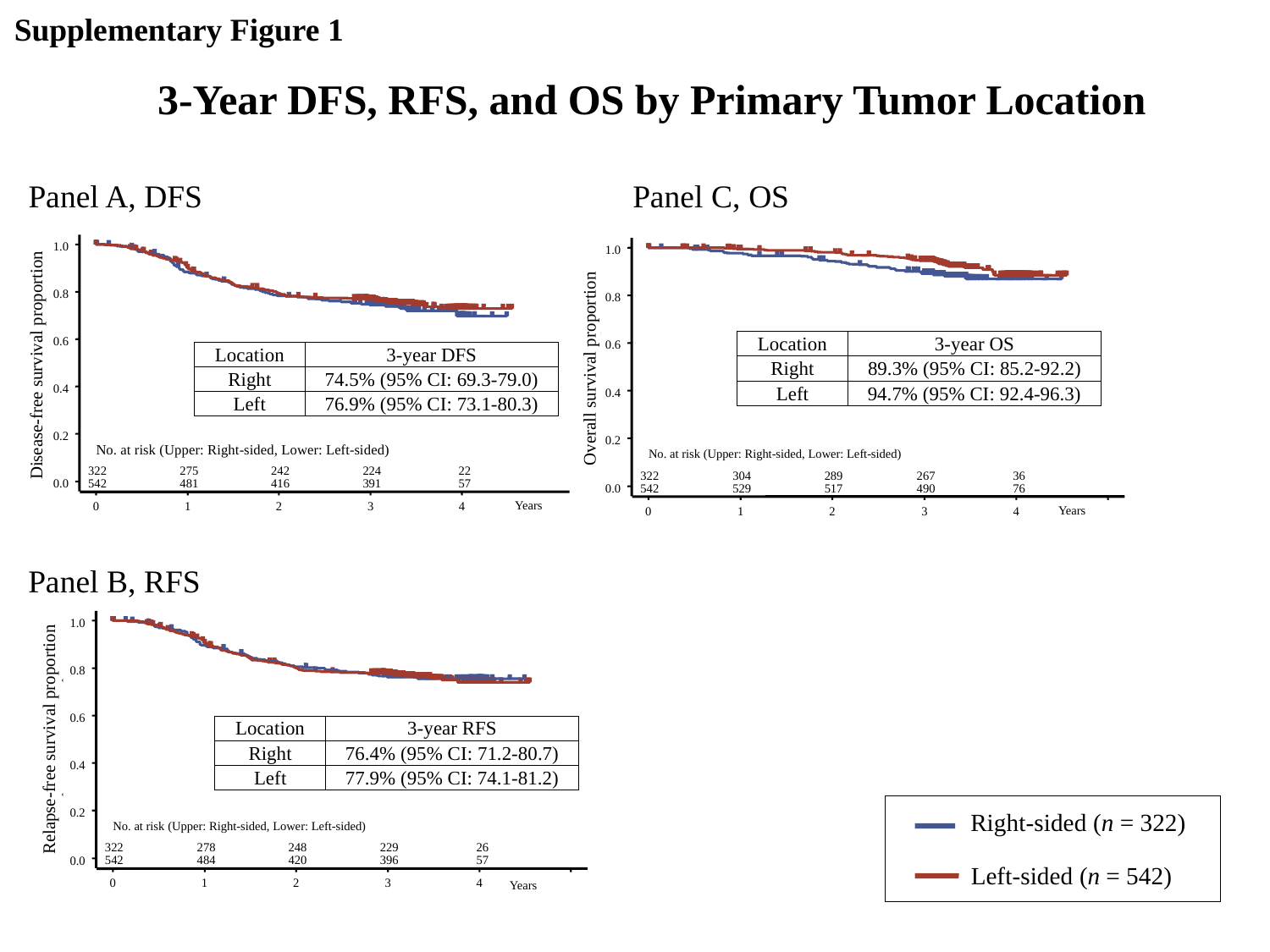

Supplementary Figure 1
3-Year DFS, RFS, and OS by Primary Tumor Location
Panel A, DFS
Panel C, OS
1.0
0.8
0.6
Disease-Free Survival Proportion
0.4
0.2
No. at risk (Upper: Right-sided, Lower: Left-sided)
322
275
242
224
 22
542
481
416
391
 57
0.0
Years
0
1
2
3
4
Disease-free survival proportion
1.0
0.8
0.6
Overall Survival Proportion
0.4
0.2
0.0
Years
0
1
2
3
4
No. at risk (Upper: Right-sided, Lower: Left-sided)
322
304
289
267
 36
542
529
517
490
 76
Overall survival proportion
| Location | 3-year OS |
| --- | --- |
| Right | 89.3% (95% CI: 85.2-92.2) |
| Left | 94.7% (95% CI: 92.4-96.3) |
| Location | 3-year DFS |
| --- | --- |
| Right | 74.5% (95% CI: 69.3-79.0) |
| Left | 76.9% (95% CI: 73.1-80.3) |
Panel B, RFS
1.0
0.8
0.6
Relapse-Free Survival Proportion
0.4
0.2
No. at risk (Upper: Right-sided, Lower: Left-sided)
322
278
248
229
 26
542
484
420
396
 57
0.0
0
1
2
3
4
Years
Relapse-free survival proportion
| Location | 3-year RFS |
| --- | --- |
| Right | 76.4% (95% CI: 71.2-80.7) |
| Left | 77.9% (95% CI: 74.1-81.2) |
Right-sided (n = 322)
Left-sided (n = 542)
