# Supplementary material for: JOIN trial: treatment outcome and recovery status of peripheral sensory neuropathy during a 3-year follow-up in patients receiving modified FOLFOX6 as adjuvant treatment for stage II/III colon cancer
Source: Cancer Chemother Pharmacol. 2019 Sep 23;84(6):1269–77. doi: 10.1007/s00280-019-03957-5 (PMC6820589; doi:10.1007/s00280-019-03957-5)
Supplement: Supplementary file 2 — Supplementary material 2 (PPTX 211 kb). Recovery status of each grade PSN (from a grade 1, b grade 2 and c grade 3) during study treatment during follow-up periods. PSN peripheral sensory neuropathy, trt treatment [file 280_2019_3957_MOESM2_ESM.pptx]

## Slide 1
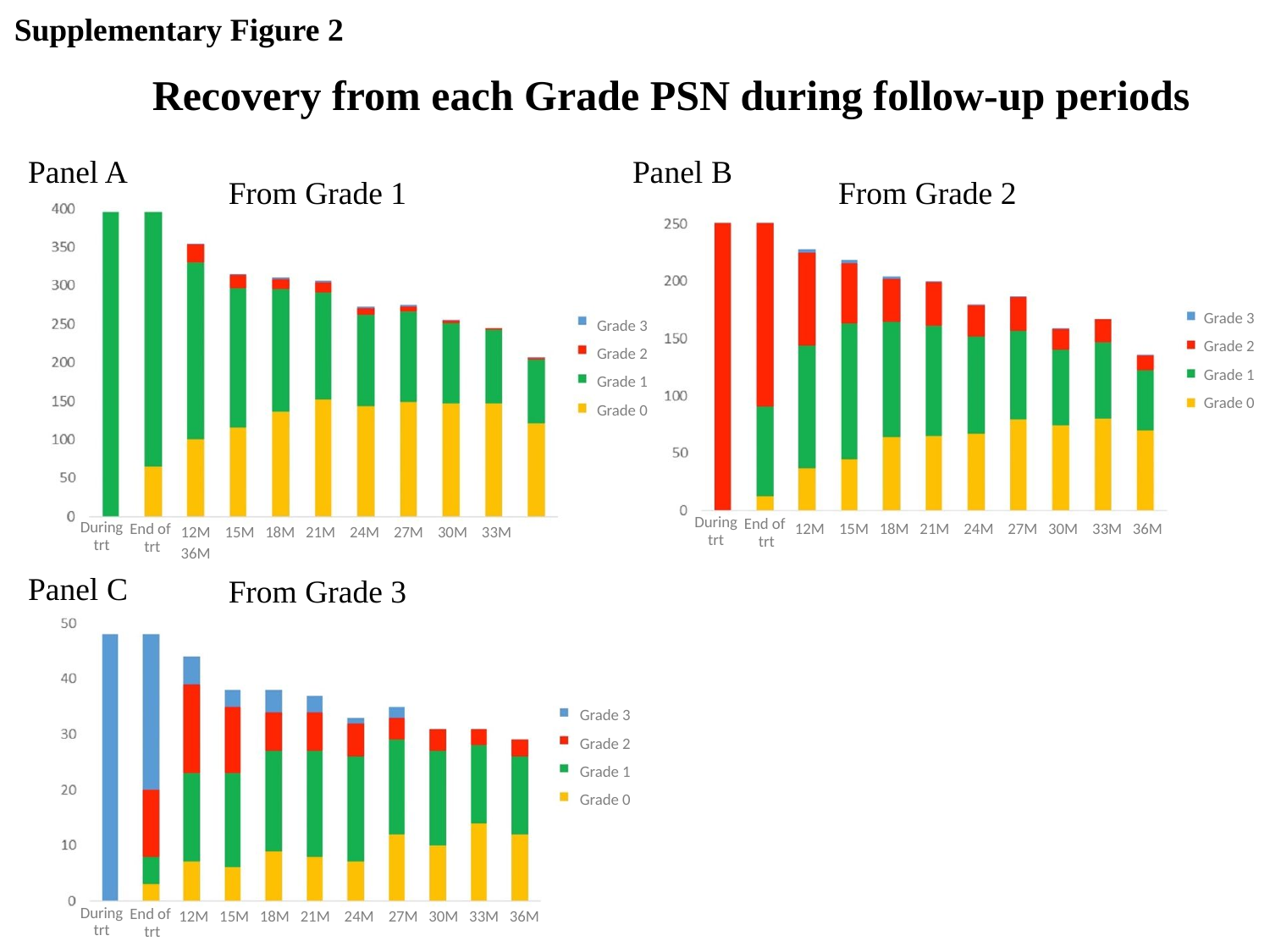

Supplementary Figure 2
Recovery from each Grade PSN during follow-up periods
Panel A
Panel B
From Grade 1
From Grade 2
Grade 3
Grade 2
Grade 1
Grade 0
During trt
End of trt
12M 15M 18M 21M 24M 27M 30M 33M 36M
Grade 3
Grade 2
Grade 1
Grade 0
During trt
End of trt
12M 15M 18M 21M 24M 27M 30M 33M 36M
Panel C
From Grade 3
Grade 3
Grade 2
Grade 1
Grade 0
During trt
12M 15M 18M 21M 24M 27M 30M 33M 36M
End of trt
