# Supplementary material for: JOIN trial: treatment outcome and recovery status of peripheral sensory neuropathy during a 3-year follow-up in patients receiving modified FOLFOX6 as adjuvant treatment for stage II/III colon cancer
Source: Cancer Chemother Pharmacol. 2019 Sep 23;84(6):1269–77. doi: 10.1007/s00280-019-03957-5 (PMC6820589; doi:10.1007/s00280-019-03957-5)
Supplement: Supplementary file 3 — Supplementary material 3 (DOCX 12 kb) [file 280_2019_3957_MOESM3_ESM.docx]

**Supplementary Table.** Cross-trial comparison of efficacy outcome across trials

| Trial Name | Regimen | Stage | N | 3y-DFS | 3y-OS |
| --- | --- | --- | --- | --- | --- |
| **JOIN** | **mFOLFOX6** | **II / III** | **864** | **76.1%** | **92.7%** |
| MOSAIC*^4^ | FOLFOX4 | II / III | 1,123 | 78.2% | 87.7% |
|  | FL (LV5FU2) | II / III | 1,123 | 72.9% | 86.6% |
| NSABP C-08*^8^ | mFOLFOX6+BV | II / III | 1,334 | 77.4% | NR |
|  | mFOLFOX6 | II / III | 1,338 | 75.5% | NR |
| JFMC 33^17^ | UFT / LV (6M) | IIB / III | 526 | 73% | 95% |
|  | UFT / LV (18M) | IIB / III | 526 | 74% | 95% |
| JFMC 37^18^ | Cape (6M) | III | 654 | 70.0% | NR |
|  | Cape (12M) | III | 650 | 75.3% | NR |
| ACTS-CC^19^ | UFT / LV | III | 760 | 72.5% | 92.7% |
|  | S-1 | III | 758 | 75.5% | 93.6% |
| JCOG 0205^20^ | UFT / LV | III | 546 | 77.8% | 93.9% |
|  | FL (RPMI) | III | 546 | 79.3% | 94.5% |
| JCOG 0910 (CAPS)^21^ | Cape | III | 782 | 81.7% | 96.3% |
|  | S-1 | III | 782 | 78.3% | 95.4% |
| SACURA^22^ | UFT | II | 985 | 86.2% | 98.0% |
|  | Surgery alone | II | 997 | 83.1% | 97.8% |
| Abbreviations: NR, not reported; FL, infusional 5FU plus leucovorin; Cape, capecitabine; M, months; RPMI, Roswell Park Memorial Institution; BV, bevacizumab; LV5FU2, infusional and bolus 5FU plus leucovorin; * Western trials | | | | | |
